# The strains recommended for use in the bacterial reverse mutation test (OECD guideline 471) can be certified as non-genetically modified organisms

**DOI:** 10.1186/s41021-016-0030-3

**Published:** 2016-01-22

**Authors:** Kei-ichi Sugiyama, Masami Yamada, Takumi Awogi, Atsushi Hakura

**Affiliations:** Division of Genetics and Mutagenesis, National Institute of Health Sciences, 1-18-1 Kamiyoga, Setagaya-ku, Tokyo, 158-8501 Japan; Drug Safety Research Center, Tokushima Research Institute, Otsuka Pharmaceutical Co., Ltd., 463-10 Kagasuno, Kawauchi-cho, Tokushima, 771-0192 Japan; Tsukuba Drug Safety, Eisai Co., Ltd., 5-1-3 Tokodai, Tsukuba-shi, Ibaraki 300-2635 Japan

**Keywords:** Bacterial reverse mutation test, Genetically modified organisms, Biodiversity, Natural occurrence, Self-cloning, pKM101, pAQ1

## Abstract

The bacterial reverse mutation test, commonly called Ames test, is used worldwide. In Japan, the genetically modified organisms (GMOs) are regulated under the Cartagena Domestic Law, and organisms obtained by self-cloning and/or natural occurrence would be exempted from the law case by case. The strains *of Salmonella typhimurium* and *Escherichia coli* recommended for use in the bacterial reverse mutation test (OECD guideline 471), have been considered as non-GMOs because they can be constructed by self-cloning or naturally occurring bacterial strains, or do not disturb the biological diversity. The present article explains the reasons why these tester strains should be classified as non-GMOs.

## Definition of genetically modified organisms

Genetically modified organisms (GMOs) are defined as an organism “in which the genetic material (DNA) has been altered in a way that does not occur naturally by mating and/or natural recombination, without using modern recombinant DNA technology” [1]. Accordingly, organisms are considered to be a non-GMO if they are made by the transfer of genetic material through bacterial conjugation between same/different species. For example, the transfer of the antibiotic resistance genes naturally occurs by bacterial conjugation in a broad host range [2]. It is also known that bacteria gain the property of antibiotic resistance through the genetic mutations and horizontal transfer of the antibiotic resistance genes under selective pressures [3]. Therefore, non-GMOs are not considered to disturb the biological diversity.

### Gene mutations of the Ames tester strains recommended in OECD guideline 471

The strains which are used in the bacterial reverse mutation test (OECD guideline 471) [4] are derivatives of *S. enterica* serovar Typhimurium (*S. typhimurium*) LT2 or *E. coli* B strain [5–7]. All the Ames tester strains recommended for use in the bacterial reverse mutation test are listed in Table 1. The *Salmonella* tester strains harbor different mutations (*hisD3052, hisG46, hisC3076, hisG428, hisD6610* and *hisO1242*) in the genes of the histidine operon of *S. typhimurium*. The *Salmonella* strains originated from *S. typhimurium* LT2 are histidine auxotrophs which are the result from treatment with mutagens or radiation [5, 6, 8–13]. In addition, the all *Salmonella* tester strains carry an *rfa* (deep rough) mutation for permeation of test chemicals, and the strains except for TA102 have a deletion mutation of *uvrB* gene to keep adducts generated with test chemicals as well as *gal, chl,* and *bio* genes [5, 6, 14]. The two tester strains of *E. coli* carry a terminating ochre mutation in the *trpE* gene as well as a *uvrA* mutation [15, 16]. Thus, the genetic background changes (mutations) in the Ames tester strains can naturally occur without using modern recombinant DNA technology.

**Table 1:** Strains recommended for use in the bacterial reverse mutation test (OECD guideline 471)

Strain	Orignal	Genotype	Plasmid
*S. typhimurium TA98*	*S. typhimurium* LT2	*hisD3052 rfa Δ(gal chl bio uvrB)*	pKM101
*S. typhimurium TA100*	*S. typhimurium* LT2	*hisG46 rfa Δ(gal chl bio uvrB)*	pKM101
*S. typhimurium TA1535*	*S. typhimurium* LT2	*hisG46 rfa Δ(gal chl bio uvrB)*	None
*S. typhimurium TA1537*	*S. typhimurium* LT2	*hisC3076 rfa Δ(gal chl bio uvrB)*	None
*S. typhimurium TA102*	*S. typhimurium* LT2	*hisG428 rfa galE hisΔ(G)8476*	pKM101, pAQ1
*S. typhimurium TA97/TA97a*	*S. typhimurium* LT2	*hisD6610 hisO1242 rfa Δ(gal chl bio uvrB)*	pKM101
*E. coli* WP2*uvrA*	*E. coli* B	*trpE uvrA*	None
*E. coli* WP2*uvrA*/pKM101	*E. coli* B	*trpE uvrA*	pKM101

### pKM101 plasmid can naturally occur and self-transmissible

As shown in Table 1, the five strains of *S. typhimurium* (TA98, TA100, TA102, TA97 and TA97a) and one strain of *E. coli* (WP2*uvrA*/pKM101) harbor plasmid pKM101. Plasmid pKM101 carries an ampicillin resistance gene and *mucAB* genes encoding analogs of UmuD/C proteins of *E. coli*, which are involved in error-prone DNA repair [6, 17]. pKM101 (35.4 kb) is derived from its clinically isolated parent R46 plasmid by an in vivo 14-kb deletion [18]. R46 plasmid contains four drug-resistance genes, while pKM101 dose not contain the other three drug-resistance genes with the exception of the ampicillin resistance gene [19, 20]. In addition, since R plasmids have a self-transmissible nature, pKM101 is normally present in the members of the family Enterobacteriaceae including the genera *Salmonella* and *Escherichia* [6, 17]. Taken together, plasmid pKM101 is considered to be a derivative of a naturally occurring plasmid, and self-transmittable.

### pAQ1 plasmid in the Ames tester strains does not disturb the biological diversity

The *S. typhimurium* TA102 strain harbors plasmids pAQ1 in addition to pKM101. The pAQ1 is a derivative of pBR322 and carries the target DNA sequence for reversion, *hisG428*, a part of the histidine biosynthetic operon originated from *S. typhimurium*. Thus, *hisG428* is a self-cloned gene. The vector pBR322 consists of the following DNA segments assembled *in vitro*; the tetracycline resistance gene, ampicillin resistance gene, and the replicator regions derived from colicin plasmid, pMB1 [21, 22]. The two drug resistance genes are derived from transposons, *Tn*10 and *Tn*3, respectively [22, 23]. Transposons are known to be transferred between related bacteria [24]. So, the drug resistance genes can be naturally introduced into the genome of *S. typhimurium*. The *hisG428* gene is inserted into the ampicillin resistance gene. It was also reported that *S. typhimurium* LT2 strains are inherently non-colicinogenic, but the strain was shown to have an ability to receive colicin plasmids from *E. coli* through conjugation [17, 25, 26]. Thus *S. typhimurium* has a possibility to have the plasmid pMB1 as well as toxin colicin naturally in their cells even without introducing pAQ1. Therefore, the introduction of pAQ1 into the Ames tester strains does not disturb the biological diversity of *S. typhimurium*, and pAQ1 plasmid can be generated *via* self-cloning technology and transferred to *S. typhimurium* LT2.

## Conclusion

In Japan, the Cartagena Domestic Law regulates living organisms resulting from modern biotechnology including recombinant DNA technology, and probable exemptions for microorganisms obtained by self-cloning and/or “natural occurrence” are assessed and decided case by case (for each produced organism) [27, 28]. Based on the following stated reasons, we conclude that all the Ames tester strains recommended for use in the bacterial mutation test [4] can be certified as non-GMOs;

1. Genetic backgrounds of the nine strains recommended for use in the bacterial mutation test [4] can be generated spontaneously, or by radiation or chemicals.
2. pKM101 harbored in the tester strains TA97, TA97a, TA98, TA100, TA102, and WP2*uvrA*/pKM101 is a naturally occurring plasmid and self-transmittable.
3. pAQ1 plasmid which the strain TA102 carries, can be generated via self-cloning technology and transferred to *S. typhimurium* LT2 by conjugation.
